# Aspirin desensitization in nonsteroidal anti-inflammatory exacerbated respiratory disease: The first prospective cohort in Chile

**DOI:** 10.3389/falgy.2022.951323

**Published:** 2023-02-01

**Authors:** María Josefina Siña, Felipe Valdés, Úrsula Zelada, María Teresa Tagle, Rolando Campillay, Daniela Sandoval, Pablo Herrera, Carla Bastías

**Affiliations:** ^1^ Department of Pediatrics, Immunology Section, Clínica Universidad de los Andes, Santiago, Chile; ^2^ Department of Internal Medicine, Immunology Section, Barros Luco Trudeau Healthcare Complex, Santiago, Chile; ^3^ Faculty of Medicine, University of Chile, Santiago, Chile; ^4^ Department of Otorhinolaryngology, Barros Luco Trudeau Healthcare Complex, Santiago, Chile

**Keywords:** nonallergic hypersensitivity, asthma, nasal polyps, Samter triad, anti-inflammatory agents, aspirin desensitization

## Abstract

**Background:**

Nonsteroidal anti-inflammatory exacerbated respiratory disease (N-ERD) is characterized by the Samter triad: chronic rhinosinusitis with nasal polyps, asthma, and nonallergic hypersensitivity to NSAIDs. Its diagnosis is based on a complete clinical history and an aspirin (ASA) challenge test. Medical treatments include biological drugs and ASA desensitization.

**Objective:**

This study aims to evaluate the clinical response of patients with N-ERD undergoing functional endoscopic surgery (FES), followed by ASA desensitization and maintenance treatment, being the first prospective cohort study carried out in Chile.

**Methods:**

We conducted 1-year follow-up of 12 patients with N-ERD treated with FES, desensitization, and maintenance with ASA. For each control, the medication score, sinonasal symptomatology (SNOT-22), PEF (peak expiratory flow), nasal polyposis (Lildholdt score), and the appearance of adverse effects were recorded. Computed tomography (CT) of the paranasal cavities was performed at baseline and at the 12-month follow-up to calculate the Lund–Mackay score.

**Results:**

Patients presented a reduction of SNOT-22 after the FES, which was maintained at 12 months (*p* = 0.002); the symptoms that showed the greatest reduction were feeling embarrassed and nasal obstruction. The Lildholdt score was also significantly reduced (*p* = 0.001); in only three patients, the nasal polyps recurred, and all were small. The PEF showed a slight nonsignificant increase of 3.3%. In total, 75% of patients had an adverse effect, the most frequent being abdominal pain (66.7%), but none of the 12 patients required discontinuation of aspirin treatment in 1-year follow-up. The Lund–Mackay score had a significant reduction of 6.6 points (*p* < 0.001).

**Conclusion:**

ASA desensitization is safe and effective in reducing upper and lower respiratory symptoms in patients with N-ERD and delays the reappearance of nasal polyps, although it is not exempt from adverse effects, with the vast majority being mild**.**

## Introduction

Nonsteroidal anti-inflammatory drugs (NSAIDs) are the most widely used group of drugs worldwide, and that is why around 25% of adverse drug reactions are attributed to this group (1). These reactions can be categorized as pseudoallergic or nonallergic hypersensitivity and, less frequently, truly allergic reactions against some component of their structure (2).

Within pseudoallergic reactions, we can find NSAID-exacerbated respiratory disease (N-ERD). Its prevalence ranges between 0.6% and 2.5% in the general population; it usually presents during the third and fourth decades of life, characterized by the clinical triad of chronic eosinophilic rhinosinusitis with nasal polyps, late-onset asthma that is usually severe, and nonallergic hypersensitivity to NSAIDs that manifests itself 30–180 min after taking these drugs when patients start or exacerbate respiratory (48%–88%) and nonrespiratory (8%–20%) symptoms (3–5).

Historically, the gold standard in the diagnosis of N-ERD has been the aspirin (ASA) challenge test. The oral challenge test has the best performance and predictive value, with a sensitivity ranging from 80% to 89% and a specificity of 93%, which makes it the most widely used route in clinical practice (6).

Available medical treatments include biological drugs and ASA desensitization. Biologics have proven to be effective and safe; however, their use is very limited due to their high cost (7). Desensitization consists of progressive administration of increasing doses of a drug to induce transient tolerance to it (8). It has shown multiple benefits in N-ERD patients such as improved quality of life, improved nasal and asthma symptoms, total or partial recovery of smell, reduced formation of new polyps, decreased number of episodes of sinusitis, and decreased need for corticosteroids (4, 9).

Adverse effects from long-term use of ASA range between 8% and 23%. The most frequent are abdominal pain and bleeding (nasal, bronchial, bladder mucosa, skin, and gastrointestinal tract) (8). Since this procedure carries risks, it should be considered in patients with poor control of symptoms, reappearance of polyps after surgical treatments, frequent use of corticosteroids, and in those who take NSAIDs for the management of comorbidities. It is contraindicated in pregnant patients, patients with unstable asthma, and patients with duodenal ulcers or coagulation disorders (4).

In Chile, until date, there have been no studies on N-ERD population response to ASA desensitization. The objective of this study was to evaluate the clinical response through symptoms score, use of medications, endoscopic evaluation of polyps, and immunological changes of paranasal sinus of Chilean patients with N-ERD undergoing functional endoscopic surgery (FES), followed by ASA desensitization and maintenance treatment after 1 year of follow-up, being the first prospective cohort study carried out in our country.

## Methods

### Study design and population

This is an experimental and prospective cohort study, which included 46 patients diagnosed with N-ERD treated in the Otorhinolaryngology Service and the Immunology section of the Barros Luco Assistance Complex in Santiago de Chile between November 2015 and November 2020.

Patients included were adults (18–60 years old), clinically diagnosed with chronic rhinosinusitis and nasal polyps supported by nasofibrosocopy and computed tomography of paranasal cavities, according to the EPOS 2021 Clinical Guideline (10); asthma confirmed by symptoms and spirometry, according to the GINA Clinical Guide used at the time of enrollment (11); a history of upper or lower respiratory symptoms associated with the intake of ASA and/or other NSAIDs in two or more occasions; and a positive ASA challenge. The latter was carried out using the validated protocol of the EAACI/GA2LEN Guide in 2007 (12), with a placebo prior to the first dose of ASA and peak expiratory flow (PEF) measurements every hour and in the event of the onset of symptoms. It was considered positive when obvious symptoms and signs of respiratory reactions appeared, such as runny nose, nasal congestion, sneezing, epiphora, bronchospasm (cough, wheezing, sensation of chest tightness, or dyspnea), or laryngospasm (stridor or dysphonia), or when there was a decrease of at least 20% in PEF from baseline. Those patients who did not present symptoms with a cumulative dose of 1,000 mg of ASA during the challenge were considered tolerant.

Excluded from the study were patients with anaphylaxis or severe skin reactions related to the intake of ASA or other NSAIDs; patients with decompensated asthma with forced expiratory volume in the first second (FEV1) and/or PEF less than 70% of theoretically predicted value; β-blocker users; pregnant women; patients with uncontrolled autoimmune, cardiac, hepatic, renal, hematological, digestive, urological and/or neurological disease; and patients with active neoplasia.

### Baseline measurements

Candidates for desensitization were evaluated by an immunology specialist through a survey in which demographic data, personal and family history, symptoms, exacerbating factors, and treatments performed were explored. A prick test for aero-allergens and a spirometry test were also requested. The visual analog scale (VAS) of the RSDI (RinoSinusitis Disability Index) translated into Spanish and validated in Spain was used (13), where 0 represents no affectation and 10 represents unbearable affectation of the quality of life. The SNOT-22 (Sino-Nasal Outcome Test 22) questionnaire was also applied by assessing 22 items on a scale from 0 to 5, granting graduation from 0 to 110 points (14, 15). This survey was validated in Chile (16). To quantify the medication requirement, a scale used in a previous study was modified (17), where the treatments for rhinitis and asthma were separated. In rhinitis, the score was divided into local and systemic treatments. For local treatments, saline nasal sprays, inhaled corticosteroids, and eye drops were considered, with one point each; for the systemic ones, oral antihistamines, oral corticosteroids, and leukotriene receptor antagonists (LTRA) were considered, with two points for each; in the case of using one in maximum dose, the score obtained for that drug was multiplied by 2. For the treatment of asthma, the use of prednisone, or equivalent, was considered intermittently (1 point), weekly (2 points), daily <20 mg (4 points), or daily ≥20 mg (6 points); the use of bronchial corticosteroids was separated into low (2 points), medium (4 points), or high (6 points) doses according to the GINA Guideline (11); at last, the use of rescue bronchodilators was separated into use ≤1 time per week (2 points), use >1 time per week (4 points), and used daily (6 points). Finally, each item mentioned was added to obtain a final score for rhinitis and asthma. All patients were evaluated by specialists in Otorhinolaryngology with inspection by nasofibroscopy. The presence or absence of nasal polyps was recorded using Lildholdt's staging, which grades polyposis from 0 to 3, with 0 = no polyps, 1 = mild polyposis, 2 = moderate polyposis, and 3 = severe polyposis. At the same time, a CT scan was requested to evaluate the initial anatomical compromise. Grading and scoring were done using the Lund–Mackay system, which assesses the degree of opacity or obstruction of six paranasal structures (maxilla, anterior ethmoid, posterior ethmoid, frontal, sphenoid, and osteomeatal complex) with a score of 0–2 for each paranasal sinus, on each side, with a maximum score of 24 points (18, 19).

### Intervention

Patients with nasal polyps underwent FES between 2 weeks and 3 months before desensitization. The objective was to remove all the visible polyps and clean the paranasal sinuses that showed opacification in tomography.

**Figure 1:**
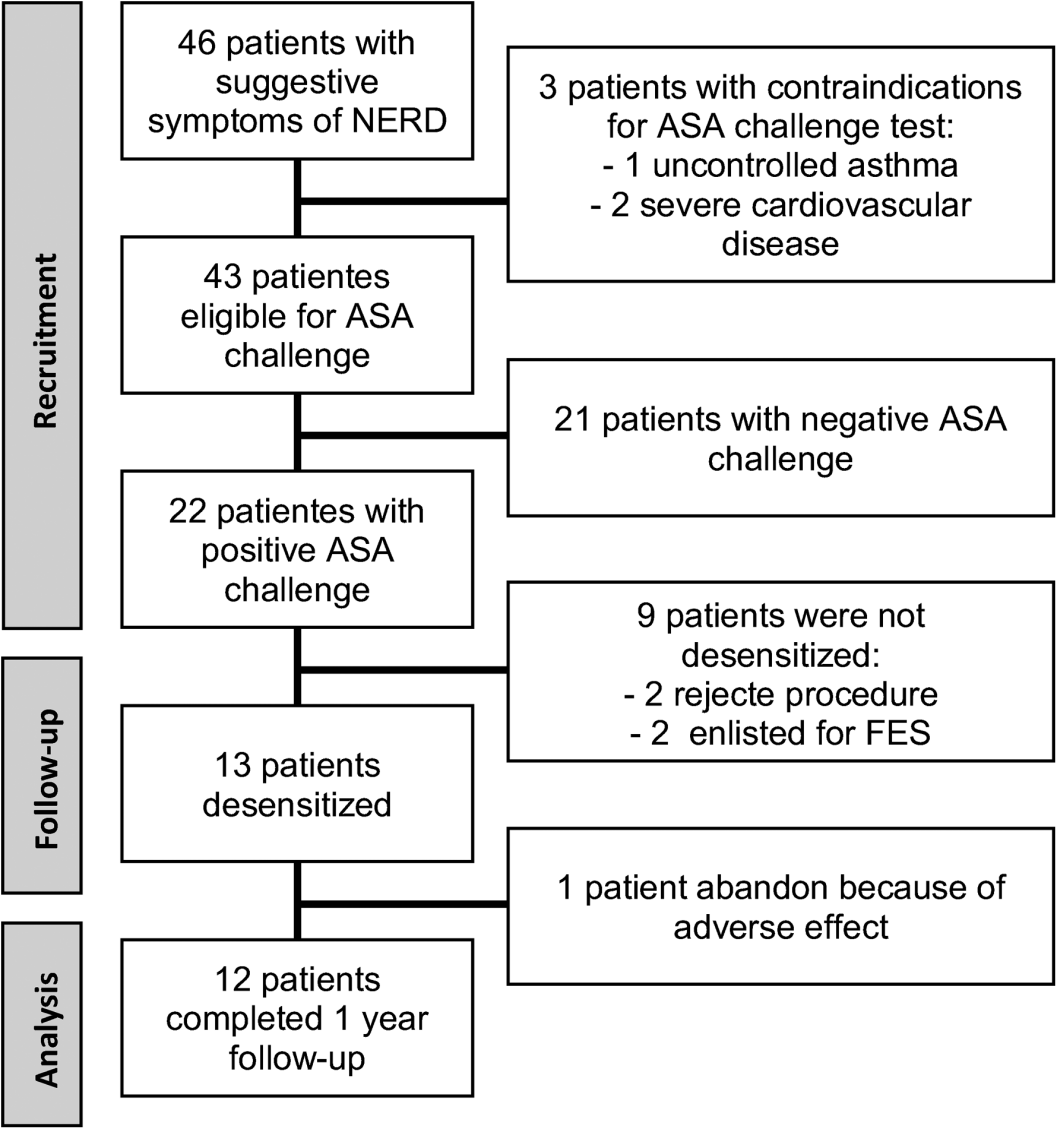
Number of patients enrolled in the study who completed the 12-month follow-up.

Desensitization was performed on hospitalized patients and based on the Scripts Clinic protocol. On day 1, three doses of ASA were administered, 20, 40, and 81 mg, each 3 h apart, and on day 2, three more doses of 81, 162, and 324 mg were administered with the same schedule to achieve a total cumulative dose of 708 mg of ASA. In the case of presenting a reaction at any step, treatment according to the symptom was indicated, and once resolved, the same dose that generated the reaction was recorded, continuing the protocol. They were discharged asymptomatic and with an indication to maintain treatment with ASA and omeprazole 20 mg every 12 h orally indefinitely. Regarding the maintenance dose, the first two desensitized patients were given ASA 650 mg every 12 h. One of them discontinued treatment immediately after desensitization due to adverse effects; so, based on the literature available to that date, it was decided to change the maintenance dose to 325 mg of ASA every 12 h in patients enrolled after this event, and this patient was not included in the follow-up results (Figure 1).

### Follow-up

Patients were controlled at 1, 3, 6, 9, and 12 months after desensitization. In each control, the maintenance dose of ASA was recorded; if there was discontinuation of treatment, its causes, adverse effects, and adjuvant treatments (inhaled and oral corticosteroids, β2-agonists, LTRA, omeprazole, antihistamines, among others) was recorded. In each control, PEF was measured, the SNOT-22 questionnaire was applied, and a review was performed with nasofibroscopy to assess the recurrence of polyps according to the Lildholdt scale. At the 12-month follow-up, a new CT scan was requested to calculate the Lund–Mackay score.

“Pre-T” was considered to be the measurements prior to the FES, as “T0” is the time of desensitization and “T1,” “T3,” “T6,” “T9,” and “T12” correspond to the follow-up controls at 1, 3, 6, 9, and 12 months after desensitization.

### Ethical considerations

This study was approved by the Ethics and Research Committee of the Barros Luco Trudeau Healthcare Complex (Memo N° 273/2015 and 555/2019). Each participant has read and signed informed consent.

### Statistical analysis

An exploratory analysis of continuous variables obtained at baseline and follow-up was performed; the Shapiro–Wilk test was applied to verify the normality of the distribution. The parametric variables were presented as means ± standard deviations variables, and nonparametric variables were presented as medians and minimum and maximum values . Categorical variables were presented as absolute and relative frequencies.

To determine the difference between the data obtained at the different follow-up times and data of baseline measurements, the Student *t*-test was used for related samples in the case of those variables in which the normal distribution was verified and the Wilcoxon test was used in the case of the nonparametric variables. It was considered a significant difference when the *p* value was <0.05.

## Results

Of the 46 patients with suggestive symptoms of N-ERD, 3 had contraindications to performing the oral ASA challenge test and 21 had a negative result. Of the 22 positive challenge patients, the median dose for the reaction was 117 mg (188 mg of cumulative ASA). Thirteen patients were desensitized, and 12 of them completed the 12-month follow-up (Figure 1). A post hoc sample size estimation was performed using GPower 3.1 software, which gave a statistical power of 0.88 using the comparison of the SNOT-22 score of PreT–T6 and 0.73 using a comparison of the SNOT-22 score of PreT–T12.

At the beginning of the study, all participants were in clinically stable conditions, with FEV1 greater than 85% of the estimate, the median SNOT-22 score at the time of diagnosis of 81 points, and the Lildholdlt score of 3. The CT scan showed an average Lund–Mackay score of 20.75 ± 2.89 points. Other baseline characteristics of these patients are presented in Table 1.

**Table 1:** Baseline characteristics of patients with N-ERD desensitized to aspirin and who completed 12 months of follow-up.

Variable	Patients (*n* = 12)
Age (years)	49 (20–65)
Sex (female/male ratio)	7/5
Symptoms evolution time (years)	10.30 ± 8.57
Age of onset of symptoms	35.60 ± 14.13
History of sinonasal surgery (yes/no)	6/6
Number of previous sinonasal surgeries	1.08 ± 1.5
Family history of rhinitis	3 (25.00)
Family history of asthma	4 (33.33)
Family history of N-ERD	1 (8.33)
Worsening of symptoms with alcohol	6 (50)
Worsening of symptoms with herbal tea	2 (16.67)
SNOT-22 score	81 (14–97)
Lildholdlt score	3 (2–3)
Lund–Mackay score	20.58 ± 2.89
Estimated % PEF	86.9 ± 13.7
Rhinitis medication score	6.83 ± 3.19
Asthma medication score	8.92 ± 4.72
Use of nasal corticosteroids	12 (100.00)
Use of antihistamines H1	11 (91.67)
Use of inhaled corticosteroids	11 (91.67)
Use of systemic corticosteroids	10 (83.33)
Use of saline nasal spray	10 (83.33)

Values are expressed as mean ± standard deviation or median (min – max) or *n* (%).

When evaluating sensitization to aeroallergens, 75% had any sensitization, 16.7% had sensitization to mites, 16.7% had sensitization to mites and pollens, and 41.7% had sensitization to pollens. Regarding the triggering factors for worsening of symptoms, the most frequent was alcohol, which worsened the symptoms in 50% of the patients, followed by herbal tea in 16.7%.

According to the basal symptoms, all patients reported nasal obstruction and olfactory alterations; 92% reported runny nose; 75% reported nasal itching, posterior discharge, and dysphonia; and 67% reported cough. Halitosis, odontalgia, odynophagia, abdominal pain, and urticaria were reported with less than 50% frequency. Regarding the quality of life evaluated by RSDI, eight patients rated their problem as unbearable (10 points), three evaluated it with a score of 9, and one ranked it with a score of 6.

During follow-up, nine patients (75%) presented an adverse reaction to ASA treatment, with the most frequent being abdominal pain in eight patients (66.67%) and epistaxis in five patients (41.67%). The presence of ecchymoses, menometrorrhagia, and urticaria was reported by three patients.

**Figure 2:**
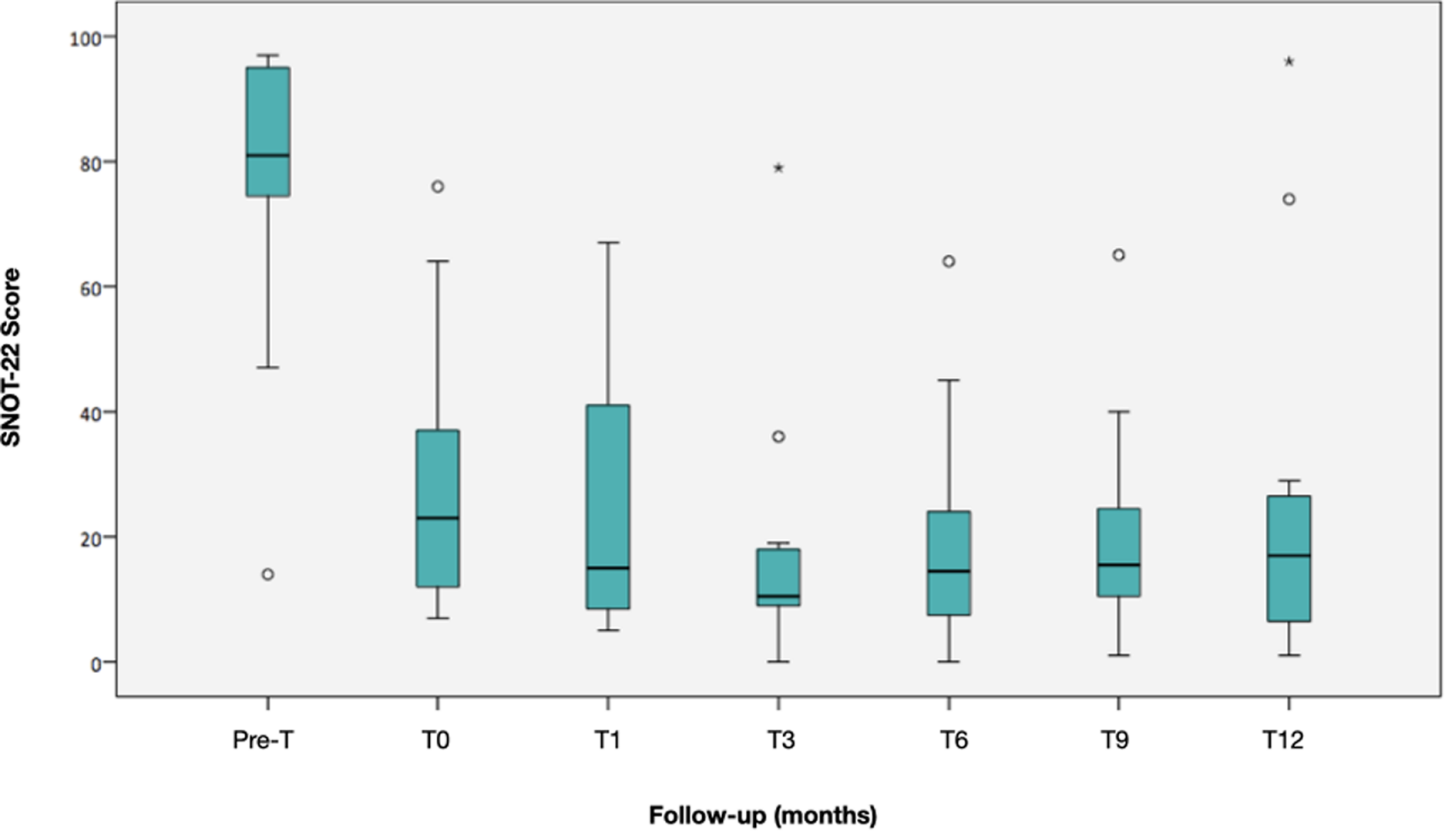
Evolution of the SNOT-22 median during follow-up. The boxes represent the 25th and 75th percentiles of the data. The lines inside the box, the median, and a whisker going to the maximum and minimum, but not more than 1.5 times the interquartile range (IQR) from the top and bottom of the box, respectively. Any value beyond the whiskers is considered an outlier and is represented by a circle. Two patients did not respond to desensitization at 12 months.

**Figure 3:**
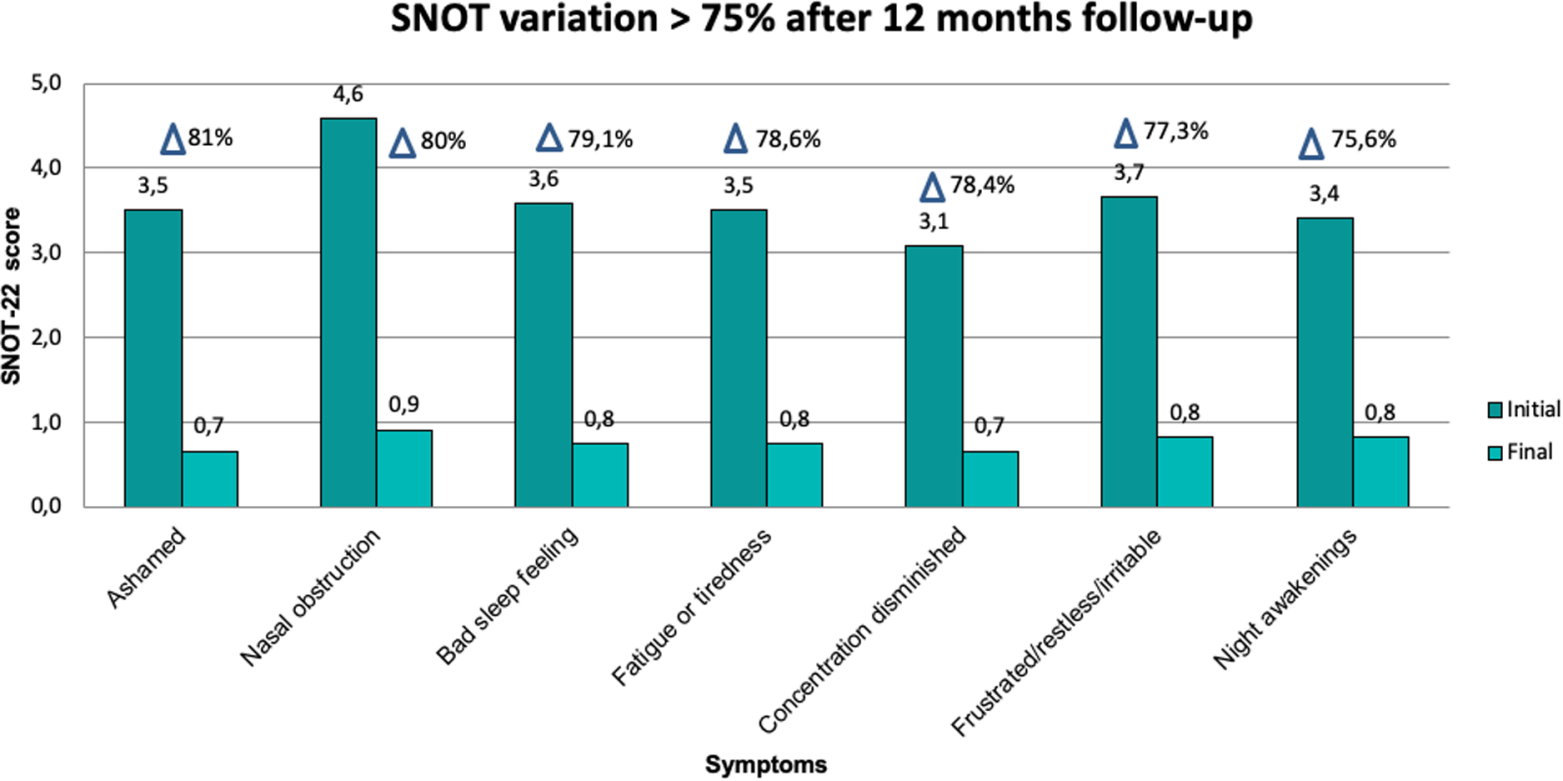
SNOT-22 variables with greater magnitude of change (>75%) before and after AAS desensitization.

Changes observed during follow-up are presented in Table 2. The SNOT-22 score showed a statistically significant reduction of 58 points in the median value after surgery (*p* = 0.003), which was maintained throughout the follow-up with a reduction of 64 points at 12 months compared to the baseline measurement (*p* = 0.002) (Figure 2). The symptoms detected through SNOT-22 and those presenting the greatest magnitude of change after desensitization are presented in Figure 3.

**Table 2:** Evolution of the clinical response of patients undergoing desensitization to ASA and comparison of each follow-up time with respect to the baseline measurement.

	Pre_T	T0	T1	T3	T6	T9	T12
SNOT-22 total	81	23	15	10.5	14.5	15.5	17
	(14–97)	(7–76)^a^	(5–67)^a^	(0–79)^a^	(0–64)^a^	(1–65)^a^	(1–96)^a^
Lildholdt	3	–	0	0	0	0	0
	(2–3)		(0–1)^a^	(0–1)^a^	(0–1)^a^	(0–2)^a^	(0–2)^a^
% PEF	86.9 ± 13.8	–	90.2 ± 18.2	89.4 ± 15.9	84.6 ± 17.7	90.9 ± 18.0	90.5 ± 18.1
Lund–MacKay	20.57 ± 2.89	–	–	–	–	–	14.08 ± 4.33^b^
Rhinitis medication score	6.8 ± 3.2	–	–	–	–	–	5.4 ± 2.3
Asthma medication score	8.9 ± 4.7	–	–	–	–	–	7.3 ± 3.9^c^

*p*-values for times T0–T12 after desensitization compare estimates with Pre-T prior to surgery and aspirin desensitization.

^a^ *p* < 0.01.

^b^ *p* < 0.001.

^c^ *p* > 0.05. Values are expressed as mean ± standard deviation or median (min – max).

The evolution of nasal polyposis objectified through the Lildholdt scale presented significant statistical differences when comparing the medians of each follow-up visit with the baseline median (*p* = 0.001, *p* = 0.001, *p* = 0.001, *p* = 0.003, and *p* = 0.003, respectively). Three patients presented recurrence of polyps, one at 9 months and two at 12 months; in all of them, the polyps were grade 1.

The evolution of PEF showed a slight rise of 3.3% at 1 month of follow-up, which was not significant (*p* = 0.451). The following measurements did not show differences in relation to baseline (*p* = 0.414; *p* = 0.478; *p* = 0.199, and *p* = 0.337, respectively). The paranasal tomographic compromise evaluated through the Lund–Mackay score showed an average decrease of 6.6 ± 3.52 points at 12 months of follow-up (*p* < 0.001). Regarding the need for medication, the score also decreased, from 6.8 to 5.4 for rhinitis and from 8.9 to 7.3 for asthma, being statistically significant only for asthma (*p* = 0.019).

## Discussion

ASA desensitization is a safe and effective method that reduces upper and lower respiratory symptoms in patients with N-ERD, in addition to delaying the reappearance of nasal polyps. In the long term, patients report significant improvements in smell and rhinosinusitis; decreased frequency of asthma attacks and sinus infections; and reduced need for systemic corticosteroids, polypectomies, and sinus surgeries (20–22). In our study, we observed a drastic reduction in symptoms, which was maintained throughout the follow-up. This response is considered clinically relevant as it shows a reduction of about 10 points in the SNOT-22 score (23, 24). Although some studies report a greater reduction, their curve of reduction and evolution of SNOT-22 during follow-up were similar to this study (25–27). It should be noted that the SNOT-22 scores were high in our sample, greater than 85 points, which suggests that most of our patients have severe diseases and worse quality of life than patients from other countries (17, 25, 26).

In addition to the reduction in symptoms, various studies have shown that FES followed by desensitization and maintenance with ASA reduces the incidence of polyp reappearance (28). Cho et al. showed that the reduction in polyposis was maintained even after 30 months of follow-up (25). In our study, 3 of 12 patients presented recurrence but with small polyps and without major clinical repercussions. Overall, patients had a significant reduction in the median polyposis score, which was maintained throughout the follow-up.

The Lund–Mackay imaging score showed a significant reduction of close to 6.6 points, which is higher than the 4 points reported as clinically relevant (17). When comparing this finding with the literature, we found differences; for example, the study by Esmaelizadeh et al. showed a minor reduction, while other studies showed no reduction in this score after 6 months of desensitization (29). This could be generated by different sample sizes, follow-up times, and severity levels at the beginning of the disease.

Regarding the need for medication according to the dose, the literature shows a decrease in the daily dose of intranasal and systemic corticosteroids. One study found a significant decrease from 271.4 to 216.3 μg/day of intranasal corticosteroids and from 10.8 to 3.6 mg/day of systemic corticosteroids (20), the latter being similar to that reported by other studies (17, 30). Our results show a reduction in the medication score at 12 months postdesensitization, but it was not statistically significant for treating rhinitis. One possible explanation is that our study, being smaller, has less statistical power to determine a significant difference. It is worth mentioning that only one patient required systemic corticosteroids due to an asthma attack, compared to the ten patients who did use prednisone intermittently prior to desensitization.

Regarding lung function, we did not find significant differences when analyzing the variation that the baseline PEF had with the different follow-up times. This is similar to that described in a publication that measured PEF, which concluded that there were no changes in asthma symptoms, FEV1, PEF, or the use of rescue medications 6 months after desensitization (29). However, there are other studies that report that FEV1 after desensitization undergoes a significant increase, suggesting better control of lung function (17, 31, 32).

Regarding the safety of the treatment, 75% of our patients developed some adverse effects, with the most frequent being abdominal pain, which was managed with proton pump inhibitors like omeprazole. The incidence of adverse effects secondary to daily use of ASA ranges from 0% to 34% (33), much lower than we observed. However, all studies agree that the most frequent adverse effect is of gastrointestinal origin, such as dyspepsia, abdominal pain, and a feeling of abdominal discomfort, mostly mild (33, 34). Gastrointestinal bleeding, in general, is rare, usually without serious complications. In our results, 40% of the patients presented epistaxis and 25% reported ecchymosis and menometrorrhagia, but none of the 12 patients required discontinuation of aspirin treatment in 1-year follow-up. The presence of adverse effects implies a risk for treatment abandonment; the estimated abandonment rate is around 37%, with a range between 0% and 52% (17, 20, 22, 25, 28–30, 33, 35, 36). In our cohort, there was only one loss immediately after desensitization due to adverse effects (8.7%).

According to the characteristics of the patients with N-ERD in our study, we had a higher prevalence of women than men, slightly lower than reported (1:1.4 vs. 1:2), and an average age of onset of symptoms of 35.6 years, consistent with the literature (4, 23, 24). The anatomical compromise measured by endoscopy and images was similar to the one described by other authors (17, 25, 37). Regarding the quality of life, 7 of 10 patients reported unbearable symptoms, with a maximum score on the VAS similar to that reported by other studies (38). Regarding the personal and family history of atopy, the literature is controversial. A high rate of atopy has been described in patients with N-ERD (39); however, in some studies, it has not been considered more prevalent than in tolerant asthmatic subjects (40). In our sample, 58.3% of the patients reported a personal or family history of rhinitis or asthma, while 75% showed some type of sensitization, with sensitization to pollens being the most frequent.

The literature estimates that 77% of patients present worsening of their symptoms, with alcohol intake being the most frequent exacerbator, followed by cow's milk in 30% of patients and toothpaste in 27% (38). In our cohort, alcohol was the most frequent exacerbator, followed by herbal tea. There are no studies explaining the association with herbal teas, which could be related to the high levels of salicylates found in products such as green tea and chamomile tea (41).

Regarding treatments performed prior to desensitization, all patients had used intranasal corticosteroids and 83.3% had systemic corticosteroids, higher than those reported in the literature (82% and 69%, respectively) (20, 42). This data could also point to more severe disease in our population.

There is consensus that using LTRA may help with asthma symptoms, with little effect on nasal symptoms and polyp size. Studies showed improved respiratory function, decreased use of rescue inhalers, and improved quality of life measurements (43). In our study, 75% reported having used LTRA at some time, less than in international studies, where 86%–88% of patients use montelukast or another LTRA (38, 42), probably because of the high cost in our environment.

In our cohort, no patient previously used allergen-specific immunotherapy or biological drugs; these are underutilized in our environment due to their high cost and limited access.

In the analysis of symptoms referred by patients, 100% of them reported nasal obstruction and hyposmia/anosmia, similar to what has been found in other studies (24, 38, 44). The presence of anosmia or hyposmia is so frequent and severe that presenting a normal sense of smell practically rules out the diagnosis of N-ERD (4). Among the least reported symptoms were abdominal pain and urticaria, both in 25% of patients, similar to other studies (4, 38), and the presence of these symptoms was associated with worse tolerance to desensitization (45).

Regarding the provocation test, the median symptom-triggering dose was 117 mg (188 mg accumulated). A systematic review of the literature in 2003 published that the average trigger dose for symptoms ranged between 30 and 150 mg of ASA, with a mean of 60 mg (46). According to other authors, reactions can occur with 157.4 mg if the drug is administered every 60 min (21) and 68 mg if it is administered every 180 min (47). Possible explanations for this variation are the protocols, formulations (aspirin or effervescent tablets), and intervals between doses. It is postulated that by using shorter time intervals, the reaction may be incorrectly attributed to the next dose of aspirin due to insufficient observation time.

One limitation of our study was the small number of participants because the selection was through concurrent sampling; however, we estimated posthoc the statistical power, and it was acceptable. Other limitations were that no asthma scores were used, and we did not compare other spirometric parameters like FEV1%, FEV1/FVC, and FEF 25%–75% nor rhinomanometric measurements to evaluate the level of nasal obstruction because of limited access in our hospital.

## Conclusions

To date, our study is the first prospective cohort study conducted in Chile and the largest in Latin America analyzing the effect of desensitization and subsequent aspirin treatment in patients with N-ERD. ASA desensitization proved safe, with mild adverse events that did not prevent treatment completion. Clinical response was characterized by a drastic reduction in symptoms, reduction in the incidence of reappearance of polyposis, significant reduction in sinonasal involvement, and decrease in the need for medication, even though our patients showed a worse symptomatology pattern and greater affectation of the quality of life than that reported in similar international studies.

## Data Availability

The original contributions presented in the study are included in the article/Supplementary Material; further inquiries can be directed to the corresponding author.
